# Influence of the first wave of COVID-19 on asthma inhaler prescriptions

**DOI:** 10.1038/s41533-021-00260-w

**Published:** 2021-11-25

**Authors:** C. I. Bloom, E. Wong, K. Hickman, S. Elkin

**Affiliations:** 1 National Heart and Lung Institute, Imperial College London, Guy Scadding Building, Dovehouse Street, London, SW3 6LY UK; 2 Imperial College Healthcare NHS Trust and Imperial College London, NHLI, London, UK; 3 West Yorkshire & Harrogate Health and Care Partnership, Low Moor Medical Practice, Bradford, West Yorkshire UK

**Keywords:** Epidemiology, Epidemiology

## Abstract

In the beginning of the COVID-19 pandemic, there were major concerns regarding the huge demand for asthma inhalers. Using the primary-care medical records for 614,700 asthma patients between January and June 2020, we found that there was a substantial increase in inhalers solely in March 2020. Patients significantly associated with receiving higher inhaled corticosteroid prescriptions were younger, of higher socioeconomic status, and had milder asthma.

During the first wave of COVID-19 in the UK, there was reported a substantial surge in prescriptions for asthma inhalers such that manufacturers stated certain inhalers were out of stock and the Department of Health and Social Care issued a statement asking practitioners not to overprescribe inhalers^1^. In this same period there was a significant reduction in the number of asthma exacerbations managed within primary care^2^; one reason proposed is an increased adherence to asthma medication. Other reasons include reduced viral exposure and fear of presentation to healthcare facilities.

In this study we have used primary-care records to quantify changes in asthma prescriptions during this period, relative to non-asthma medications. We have also sought to understand which patients were more likely to receive increased prescriptions, as this could inform on whether these increased prescriptions were likely to be related to increased adherence.

A total of 614,700 asthma patients and 240,661,066 issued prescriptions were identified. All prescriptions increased during March 2020, with a peak on the 16th of March, corresponding to the dotted line in Fig. 1.

**Fig. 1:**
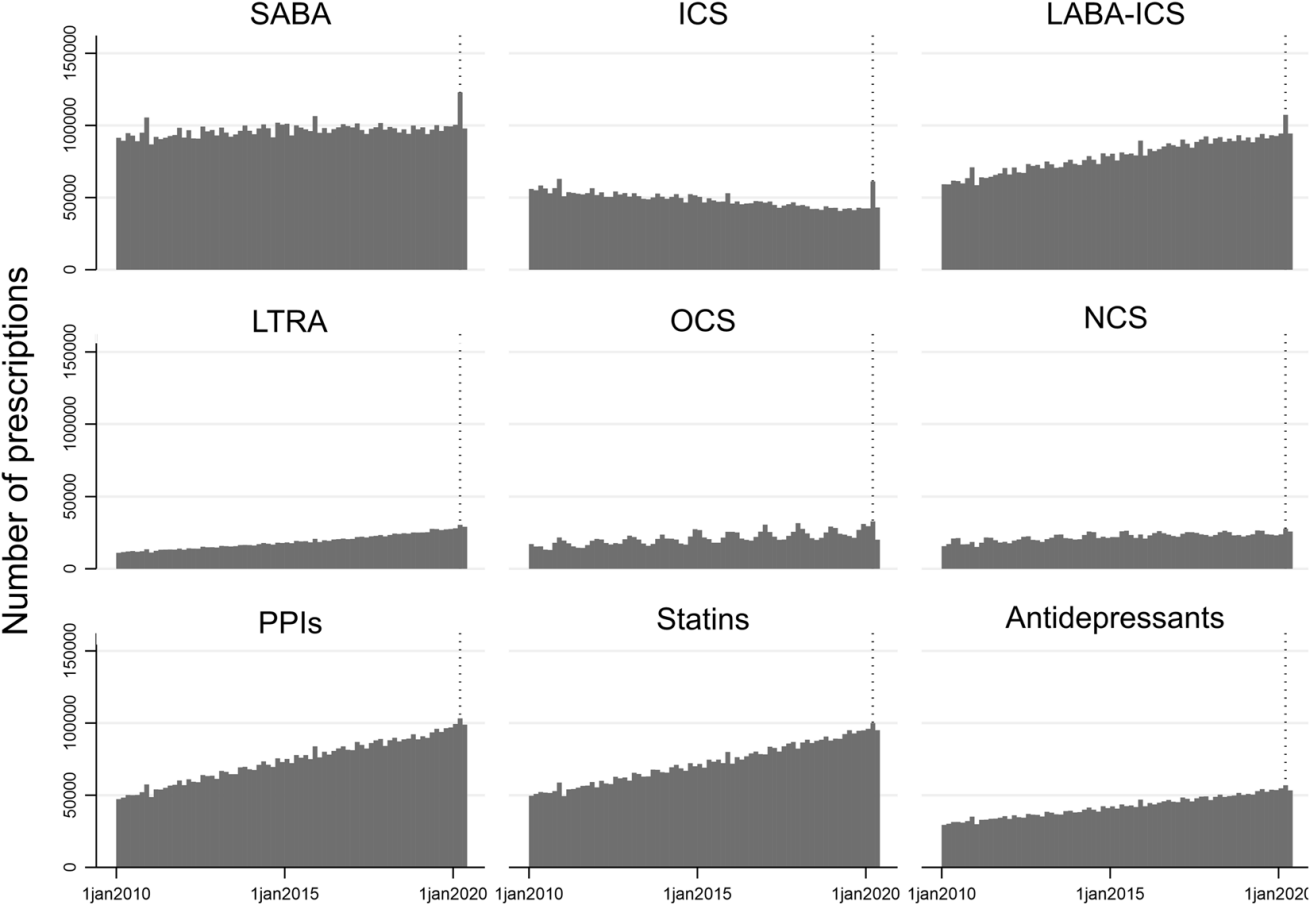
Number of daily prescriptions by drug type from 1st January 2010 until 1st June 2020. Each bar represents a day. The dotted line is placed on the date 16th March 2020.

The proportion of the following medications increased significantly in March 2020 as compared to March 2019; the largest change was seen in asthma medications: (a) ICS: 52.55% increase (2019: 3.92% (95% CI 3.85–4.00), 2020: 5.98% (95% CI 5.91–6.06), *p* < 0.0001); (b) SABA: 44.23% increase (2019: 10.31 (95% CI 10.20–10.43), 2020: 14.87% (95% CI 14.76–14.99), *p* < 0.0001); (c) OCS: 17.37% increase (2019: 1.9% (95% CI 1.85–1.95), 2020: 2.23% (95% CI 2.19–2.28), *p* < 0.0001); and (d) LABA-ICS: 14.19% increase (2019: 3.92 (95% CI 3.85–4.00), 2020: 5.98% (95% CI 5.91–6.06), *p* < 0.0001).

The following medications decreased significantly in March 2020 as compared to March 2019 (*p* < 0.0001): LTRA (0.53% decrease), antidepressants (10.15% decrease), statins (10.33% decrease), NCS (3.31% decrease), and PPIs (6.95% decrease).

The most highly prescribed ICS inhaler in March 2019 and 2020 was Clenil Modulite 100 mcg, and Fostair 100/6 was the most highly prescribed LABA-ICS in both years. There was a 212.96% increase in Clenil Modulite prescriptions in March 2020 and 170.78% increase in Fostair 100/6 prescriptions (Fig. 2). Changes in the months before and after March were considerably less; the level of change is in keeping with the mild changes that occur between calendar years.

**Fig. 2:**
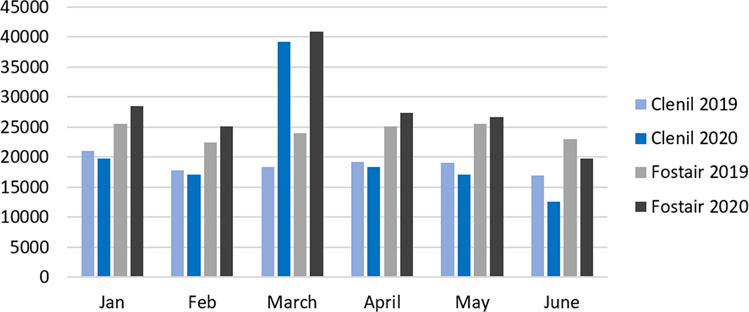
The number of prescriptions by inhaler, month and year. Comparison of the number of prescriptions in March 2019 and March 2020 for the most highly prescribed ICS (Clenil Modulite 100 mcg) and LABA-ICS (Fostair 100/6).

In all, 97,692 patients were included in the regression model. Several factors were significantly associated with an increased odds of receiving more ICS prescriptions in March 2020 than in March 2019; these included age under 50 years, not having COPD, higher socioeconomic status, having had no exacerbations in the year prior, and receiving a prescription for a low-dose ICS or receiving less than three maintenance inhalers (see Supplementary Table 1). Patient’s gender, having had a recent annual asthma review, and their number of reliever inhalers in the year prior were not significantly associated with receiving more ICS prescriptions (*p* > 0.05).

The factors significantly associated with an increase in odds of receiving more OCS prescriptions included having had an exacerbation in the year prior (adjusted OR = 5.74, 95% CI 5.39–6.12, *p* < 0.0001), having COPD (adjusted OR = 1.99, 95% CI 1.87–2.12, *p* < 0.0001), using high-dose ICS (adjusted OR = 1.43, 95% CI 1.27–1.60, *p* < 0.0001), and having at least six maintenance inhalers (adjusted OR = 1.25, 95% CI 1.19–1.35, *p* < 0.0001).

In summary, there was an increase in inhaler prescriptions, but not other commonly prescribed medications, in asthma patients during the month of March 2020, but the levels fell back to typical numbers thereafter. The peak in March coincided with the day that the government announced people with asthma should shield (16th March)^3^. The total number of prescriptions for the most frequently prescribed ICS inhaler, Clenil Modulite 100 mcg, doubled in March 2020. Asthma patients who increased their ICS prescriptions were more likely to be younger, have minimal socioeconomic deprivation, and have mild asthma (no recent exacerbation, on low-dose ICS, and have infrequent ICS prescriptions). A possible study limitation was selection bias; to capture the change in individual patient’s prescriptions beyond that occurring year-on-year, only patients with at least 5 years of inhaler data were included. It seems unlikely that the increase in prescriptions was related to increased prolonged adherence leading to a reduction in exacerbations, but adherence data for this study were not available. In contrast, the factors associated with increased OCS prescriptions were synonymous with patients who had more severe respiratory disease.

## Methods

### Data sources and variables

We used the Clinical Practice Research Datalink (CPRD) Aurum, a database of primary-care medical records covering approximately 19% of the UK^4^. A cohort of adult (≥18 years) asthma patients using a validated algorithm^5^ were identified; patients were excluded if their last asthma code was over 3 years ago or they had less than 5 years of prescription data. Medications between 1st January 2010 and 31st May 2020 were included. Asthma medications included inhaled corticosteroids (ICS), short-acting beta-agonists (SABA), combination long-acting beta-agonists and ICS (LABA-ICS), and leukotriene receptor antagonist (LTRA); non-asthma medications included the frequently prescribed medications, nasal corticosteroids (NCS), proton pump inhibitors (PPI), statins, and the three most prescribed antidepressants (fluoxetine, citalopram, and sertraline)^6^. Comparing the prescription of asthma medications to the most commonly prescribed of all medications allows assessment of the specificity of changes in asthma medications.

### Statistical analysis

Prescriptions were visualised using histograms, including all prescriptions. Thereafter, analysis was performed after removing repeat prescriptions on the same day for the same medication (attempting to reduce the prescriptions intended for stock piling). The difference in prescriptions per patient between 1st and 31st March 2019, and the same period in 2020, was reported using percentage change. Wilcoxon signed-rank test was used to confirm if there was a statistical difference, where significance was defined as *p* < 0.001. The difference in the two most frequently prescribed ICS and LABA-ICS inhalers was measured using the percentage change of the absolute prescription numbers. Multivariable logistic regression was used to estimate the association between increased ICS prescriptions (March 2019 to March 2020) and patient’s age, gender, age, socioeconomic status, COPD comorbidity, and in the year prior: exacerbations managed within primary care (defined as a short course of OCS), annual asthma review, frequency of SABA use and maintenance inhaler use (ICS or ICS-LABA), and dose of ICS (low < 800 mcg, medium, or high ≥1000 mcg).

### Ethics

The protocol for this research was approved by the Independent Scientific Advisory Committee (ISAC) for MHRA Database Research (protocol 20_096). CPRD data are already approved via a national research ethics committee for non-interventional research. Because patients cannot be identified from the data that GP practices send to CPRD, GPs do not need to seek individual written patient consent when they share the data with CPRD. However, patients can opt out of their patient information being shared for research.

### Reporting Summary

Further information on research design is available in the Nature Research Reporting Summary linked to this article.

## Supplementary information

Supplementary Information

Reporting Summary

## Data Availability

Data were obtained from a third party (CPRD) and are not publicly available. CPRD data cannot be directly shared by the researchers but are available directly from CPRD subject to standard conditions. Access to anonymised patient data from CPRD is subject to a data sharing agreement (DSA) containing the detailed terms and conditions of use following protocol approval from CPRD’s ISAC. The generated synthetic data set discussed in this paper can also be requested from CPRD subject to a DSA (https://www.cprd.com/content/synthetic-data).
