# Supplementary material for: Influence of the first wave of COVID-19 on asthma inhaler prescriptions
Source: NPJ Prim Care Respir Med. 2021 Nov 25;31:45. doi: 10.1038/s41533-021-00260-w (PMC8617286; doi:10.1038/s41533-021-00260-w)
Supplement: Supplementary file 1 — Supplementary Information [file 41533_2021_260_MOESM1_ESM.pdf]

Supplementary Table 1. *Association between patient demographics and characteristics and having an increased prescription of ICS inhalers in March 2020 as compared to March 2019.*

	Odds Ratio	95% CI	p-value
**Age**			
< 50 years		*Reference*	
≥50 years	0.79	0.76-0.82	0.00
**Gender**	1.02	0.98-1.06	0.38
**COPD comorbidity**	0.61	0.57-0.64	0.00
**Socioeconomic status**			
1		*Reference*	
2	1.17	1.09-1.24	0.00
3	1.20	1.12-1.27	0.00
4	1.24	1.16-1.32	0.00
5 (least deprived)	1.32	1.24-1.4	0.00
**Characteristics in year before**			
**Annual asthma review**	1.00	0.96-1.04	0.84
**Exacerbations**			
None		*Reference*	
≥1	0.80	0.76-0.84	0.00
**ICS dose**			
No ICS		*Reference*	
Low dose	6.72	6.01-7.52	0.00
Medium dose	2.11	1.88-2.37	0.00
High dose	0.69	0.59-0.81	0.00
**Maintenance inhalers (ICS or LABA-ICS)**			
≥6		*Reference*	
3-6	1.64	1.53-1.75	0.00
<3	2.73	2.57-2.9	0.00
None	5.48	4.84-6.2	0.00
**Reliever inhalers (SABA)**			
None		*Reference*	
1-3	1.01	0.96-1.06	0.71
≥4	0.95	0.9-1.01	0.08
